# The Role of Gut and Lung Microbiota in Susceptibility to Tuberculosis

**DOI:** 10.3390/ijerph182212220

**Published:** 2021-11-21

**Authors:** Pasquale Comberiati, Maria Di Cicco, Francesco Paravati, Umberto Pelosi, Alessandro Di Gangi, Stefania Arasi, Simona Barni, Davide Caimmi, Carla Mastrorilli, Amelia Licari, Fernanda Chiera

**Affiliations:** 1 Department of Clinical and Experimental Medicine, University of Pisa, 56126 Pisa, Italy; maria.dicicco@unipi.it (M.D.C.); diga.92@live.it (A.D.G.); 2 Allergology and Pulmonology Section, Pediatrics Unit, Pisa University Hospital, 56126 Pisa, Italy; 3 Department of Clinical Immunology and Allergology, I.M. Sechenov First Moscow State Medical University, Moscow 119991, Russia; 4 Department of Pediatrics, San Giovanni di Dio Hospital, 88900 Crotone, Italy; paravati.f@gmail.com (F.P.); fernandachiera@hotmail.it (F.C.); 5 Pediatric Unit, Santa Barbara Hospital, 09016 Iglesias, Italy; umberto.pelosi@gmail.com; 6 Area of Translational Research in Pediatric Specialities, Allergy Unit, Bambino Gesù Children’s Hospital, IRCCS, 00165 Rome, Italy; stefania.arasi@yahoo.it; 7 Allergic Unit, Department of Pediatric, Meyer Children’s Hospital, 50139 Florence, Italy; simonabarni@hotmail.com; 8 Allergy Unit, CHU de Montpellier, Université de Montpellier, 34295 Montpellier, France; davide.caimmi@gmail.com; 9 IDESP, UMR A11, Université de Montpellier, 34093 Montpellier, France; 10 Department of Pediatrics, University Hospital Consortium Corporation Polyclinic of Bari, Pediatric Hospital Giovanni XXIII, 70124 Bari, Italy; carla.mastrorilli@icloud.com; 11 Pediatric Clinic, Pediatrics Department, Policlinico San Matteo, University of Pavia, 27100 Pavia, Italy; A.Licari@smatteo.pv.it

**Keywords:** children, gut, infection, lung, microbiota, microbiome, *Mycobacterium tuberculosis*, tuberculosis

## Abstract

Tuberculosis is one of the most common infectious diseases and infectious causes of death worldwide. Over the last decades, significant research effort has been directed towards defining the understanding of the pathogenesis of tuberculosis to improve diagnosis and therapeutic options. Emerging scientific evidence indicates a possible role of the human microbiota in the pathophysiology of tuberculosis, response to therapy, clinical outcomes, and post-treatment outcomes. Although human studies on the role of the microbiota in tuberculosis are limited, published data in recent years, both from experimental and clinical studies, suggest that a better understanding of the gut–lung microbiome axis and microbiome–immune crosstalk could shed light on the specific pathogenetic mechanisms of *Mycobacterium tuberculosis* infection and identify new therapeutic targets. In this review, we address the current knowledge of the host immune responses against *Mycobacterium tuberculosis* infection, the emerging evidence on how gut and lung microbiota can modulate susceptibility to tuberculosis, the available studies on the possible use of probiotic–antibiotic combination therapy for the treatment of tuberculosis, and the knowledge gaps and future research priorities in this field.

## 1. Introduction

Tuberculosis (TB) is one of the most common infectious diseases and infectious causes of death worldwide. About 10 million new cases of TB and 1.4 million deaths from TB were observed in 2019, with 250,000 deaths due to multidrug resistance [1]. Pediatric TB accounts for approximately 10–20% of all cases, with the 0–4 age group being at higher risk of disseminated disease and mortality [1,2,3]. The prevalence of TB in the pediatric age is likely underestimated due to less specific clinical manifestations and radiological findings, lower bacillary load, and greater difficulty obtaining an adequate sample for the microbiological diagnosis than in adults [4].

TB is caused by *Mycobacterium* (M.) *tuberculosis*, an intracellular pathogen that has an airborne transmission. In most cases, primary infection with *M. tuberculosis* is asymptomatic and commonly progresses into a latent tuberculosis infection (LTBI), in which patients undergo clinical but not biological recovery, remaining infected with quiescent mycobacteria. About 5–10% of subjects with primary *M. tuberculosis* infection may immediately manifest active TB (primary TB), defined as clinical symptoms of disease, microbiological confirmation of *M. tuberculosis*, or both, or undergo clinical reactivation of the LTBI throughout life (secondary TB), because of failure to develop or maintain an effective immune response. Age, immune deficiencies, malnutrition, and bacterial load are the most important factors for the rapid replication of *M. tuberculosis* and progression to active TB [2].

Accumulating evidence suggests that the human microbiota dysbiosis could modulate susceptibility to *M. tuberculosis* infection, progression from LTBI to active TB, and response to antituberculosis therapy [5]. In this review, we address the current understanding of the host immune response against *M. tuberculosis* infection, the emerging evidence on the possible role of gut and lung microbiota in TB pathogenesis, and the available data on the possible use of probiotics in combination with standard antibiotic therapy for the treatment of TB.

## 2. Immune Response to Mycobacterium Tuberculosis

*M. tuberculosis* infection is extremely peculiar due to the unique virulence factors of this intracellular bacteria and the complex immune response triggered by this infection. Both innate and adaptive immunity play a crucial role in controlling *M. tuberculosis* infection, and their interaction contributes to the clinical manifestations of the disease. *M. tuberculosis* uses multiple immune evasion mechanisms, which can prevent the formation of an immune response capable of eradicating the infection.

### 2.1. Innate Immunity

The immune response to *M. tuberculosis* is activated by the exposure of the airway epithelium to the bacillus. Recent evidence shows that immediately after exposure, *M. tuberculosis* can infect the cells of the respiratory mucosa, which, in response, stimulate the production of some cytokines, in particular interferon-gamma (IFN-γ), by resident populations of CD8⁺ mucosal-associated T lymphocytes (MAIT) [6]. This mechanism may contribute to the clearance of *M. tuberculosis* in individuals exposed before the onset of the immune response, or it may play a role in triggering the cell-mediated immune response.

At the alveolar level, *M. tuberculosis* is engulfed by resident macrophages which aim to inhibit bacterial replication and eradicate the infection [7,8].

In this first stage, the ability to eradicate *M. tuberculosis* depends on the microbicide functionality of the alveolar macrophages and the virulence factors of the mycobacteria. *M. tuberculosis* has developed several mechanisms mediated by glycolipids and proteins of the bacterial cell wall (e.g., inhibition of the Ca2^+^/calmodulin pathway), preventing the cytotoxic activity of alveolar macrophages. This allows some mycobacteria to survive and multiply within macrophages, leading to macrophage lysis and the release of cytokines and bacterial antigens into the extracellular environment. Such mediators lead to the recruitment of other inflammatory cells, lymphocytes, monocyte-macrophages, and dendritic cells to the primary infection site. The dendritic cells then migrate to the lymph nodes to present the antigens of *M. tuberculosis* to the T-naive lymphocytes and activate the adaptive immune response [7,8,9,10].

Therefore, alveolar macrophages have a dual immunological role in *M. tuberculosis* infection: on the one hand, such immune cells are potentially able to extinguish the infection or at least contain it; on the other hand, macrophages represent the preferential habitat for *M. tuberculosis* and can become its reservoir for a rapid and uncontrolled replication, as occurs in cases of miliary TB in those individuals with immunodeficiency or genetic susceptibility [11,12].

Natura killer (NK) cells and neutrophils can also contribute to the immune response against *M. tuberculosis*, but the mechanisms by which they act are not well defined [13,14,15] Although dendritic cells are essential for activating T-cells and maintaining a balance in the inflammatory state, their role as a replicative niche for *M. tuberculosis* has recently been demonstrated [16].

### 2.2. Adaptive Immunity

The adaptive response mediated by T-lymphocytes develops approximately 2–4 weeks after mucosal exposure to *M. tuberculosis*. The crucial role of the T-mediated response in protecting against *M. tuberculosis* infection is well demonstrated by the higher risk for a severe, treatment-resistant, and fatal TB in patients with CD4+ T lymphocyte deficiency, such as those with HIV infection [10].

Multiple T cell populations contribute to the adaptive immune response against *M. tuberculosis*, particularly CD4+ T cells, CD8+ T cells, and other “unconventional” T cell populations.

Following IL-12 stimulation, CD4+ T lymphocytes differentiate into Th1 lymphocytes, which can produce large amounts of IFN-γ [8]. Under the action of IFN-γ, macrophages are activated, acquire the M1 phenotype [17], which has a greater bactericidal capacity, and accumulate in the primary infection site [8].

CD8 + T lymphocytes are also involved in protective immunity since, in addition to producing IFN-γ and other proinflammatory cytokines, they have a direct cytotoxic activity on infected macrophages and *M. tuberculosis*, thus facilitating the control of infection both in the acute and chronic phase [18].

The activation of macrophages by Th1 lymphocytes appears to be crucial for containing *M. tuberculosis* infection in specific granulomas.

A predisposition of the immune response towards the Th2 type can alter the Th1/Th17 balance and the action of cytotoxic CD8+ T lymphocytes and has been associated with a significant risk of progression of tuberculous lung injury [19,20].

In most cases, although the adaptive immune response fails to “sterilize” the primary lesion, the immune system achieves a balance between infection and containment, whereby *M. tuberculosis* enters a state of quiescence controlled by the cell-mediated immunity, establishing a clinically asymptomatic LTBI [21].

Only about 5–10% of those infected with *M. tuberculosis* develop active TB, which can occur immediately after primary infection, usually within two years of contagion, due to the inability of the immune system to contain the infection, or throughout life concomitantly with conditions that impair immune responses [21]. The altered balance between infection and immune containment can result in the resumption of the multiplication of mycobacteria and the formation of multiple granulomas, which, following a process of colliquative necrosis, can invade the bronchi, favoring air transmission of the infection to other subjects and the bloodstream, aiding the extrapulmonary dissemination of the infection.

*M. tuberculosis* infection, therefore, does not lead to the development of permanent immunity, hence the marginal role of B cells in this infection [10].

## 3. The Gut and Lung Microbiota

The gut microbiota is a complex and dynamic ecosystem that is home to more than 100 trillion commensal microorganisms. The taxonomic composition of the human gut microbiota is dominated by *Firmicutes* and *Bacteroidetes*, with slightly lower levels of *Actinobacteria* and *Proteobacteria*, and other important phyla such as *Verrucomicrobia*, *Fusobacteria*, and *Euryarchaeota* [22].

Scientific evidence suggests that the gut microbiota exerts its beneficial effects through the involvement of physiological processes such as digestion and absorption of nutrients, modulation of the immune system, and protection from pathogen invasion [23,24,25].

In recent years, accumulating evidence suggests that the composition of the intestinal microbiota in the first years of life is a determining factor for the maturation of the immune system, the maintenance of immunological tolerance, and the individual’s health during life. The composition of the intestinal microbiota changes dynamically in the first 2–3 years of life and can be influenced by both genetic factors and various environmental factors, such as the mode of delivery (cesarean or natural), the type of feeding (breastfeeding or formula), the use of antibiotics, the type of diet, the living environment, and the use of disinfectant products for hygiene [26,27,28,29].

The loss in richness and biodiversity of the microbiota, a process called “dysbiosis”, causes an alteration of its metabolic activities and has been associated with a greater susceptibility to immune-mediated disorders throughout life, such as inflammatory bowel diseases and allergic diseases, which have been on the rise for several decades [27,28,29].

Over the last few years, multiple mechanisms, not necessarily pathological-specific, have been identified that allow the gut microbiota to regulate the immune response and vice versa [26]. The functional immunological role of the intestinal microbiota is well demonstrated on “germ-free” mouse models, in which the absence of the microbiota determines the reduction of the gut-associated lymphoid tissue (GALT) and critical immunological defects in both innate and adaptive immunity [30].

Recent evidence suggests that the immunomodulatory effects of the intestinal microbiota can occur both locally and in other organs, such as the lungs, creating the so-called “gut–lung axis” [8]. Alterations of the intestinal microbiota or the metabolites produced by it have been associated with deficits in the immune response to influenza and pulmonary inflammation processes in the context of various chronic respiratory diseases [28,31,32,33,34,35].

For a long time, the lungs of the healthy individual were considered a sterile environment that could only be colonized in case of lung disease. This belief originated from some erroneous assumptions due to the methodological limitations of available microbiological tests, techniques of sampling (high risk of contamination of the sample with germs from the upper airways using the study of sputum and material taken during bronchoscopy), and natural contamination of the lower airways by inhaled material [36]. This mistaken belief had initially led to the exclusion of the lung from the *Human Microbiome Project* [37]. Since 2010, owing to next-generation genome sequencing techniques, numerous studies have shown that the lungs of healthy subjects are not sterile but are colonized by numerous microorganisms, including bacteria, viruses, and fungi [38,39,40]. These new techniques have made it possible to identify different species of bacteria in the lungs, at the phylum level (*Firmicutes*, *Bacteroides*, and *Proteobacteria*) and the genus levels (*Veillonella*, *Prevotella*, *Fusobacteria*, and *Streptococcus*, with the presence of small amounts of potential pathogens such as *Haemophilus*) and of fungi (*Aspergillus*, *Cladosporium*, *Penicillium*, and *Eurotium*) [39].

The respiratory system does not have a similar habitat in all its districts (bronchi, bronchioles, alveoli) and, therefore, the composition of the lung microbiota is influenced by a multitude of factors, including microbial immigration (microaspiration, inhalation of microorganisms, direct mucosal dispersion), microbial elimination (cough, mucociliary clearance, innate and adaptive immunity) and local growth conditions (micronutrients availability, temperature, oxygen tension, local microbial competition, concentration and activity of inflammatory cells) [41]. The balance between these factors, particularly the first two, is still considered the key driver of the composition of the lung microbiota in healthy subjects. Local factors in the healthy subject determine an unfavorable environment for the growth of bacteria and their multiplication. On the contrary, when the local environment changes, creating well-defined entities (niches), the multiplication of germs and the onset of diseases, including chronic ones, are favored [36].

Similarly to the intestinal microbiota, the composition of the lung microbiota is influenced not only by the anatomical characteristics of the lung but also by genetic and environmental factors (such as the use of medication, living on farms, number of siblings, presence of pets, cigarette smoking) [42,43,44,45,46,47,48,49,50,51]. In particular, the improper use of drugs, such as antibiotics, anti-inflammatories, and corticosteroids, can lead to significant alterations of the microbiota, which may return to the original composition, or result in a permanent alteration of either the composition or the function, or both [42,43,44,45].

The neonatal period is emerging as a crucial time window that can shape the composition and function of the lung microbiota. Recent evidence documents the presence of bacteria in the placenta, amniotic fluid, fetal membranes, and cord blood, which shows that the lung microbiota is already present at birth, thus refuting the belief that the fetal environment was sterile [46,47]. Microbial communities have been detected from the first days of life in the oral and nasopharyngeal cavities of term infants (*Staphylococcus*, *Streptococcus*, and *Moraxella*) and the respiratory tract of intubated preterm infants (*Proteobacteria*) [48]. Finally, there is also evidence that the lung microbiota changes with age and with changes in respiratory function [46,47].

### 3.1. Interactions between the Host Microbiome and Innate Immunity

Several mechanisms underlying the interaction between intestinal microbiota and innate immunity are still not all well-defined. At the level of the intestinal lumen, the action of the resident microbiota favors the production of numerous molecules binding TLRs and NOD-like receptors (NLRs), and metabolites with immunomodulating action, such as short-chain fatty acids (SCFAs), which contribute to homeostasis and the development of the intestinal immune response [26].

TLRs are involved in the defense of the host against pathogenic microorganisms, regulate the abundance and composition of the commensal intestinal microbiota, and maintain the integrity of tissues and mucous barriers. The mapping of the expression of TLRs receptors on epithelial cells of the intestinal mucosa revealed the presence of specific time–spatial patterns, with a greater variety in the mucosa of the colon than that of the small intestine, and the presence of site-specific receptors, such as the TLR-5 found on intestinal Paneth cells [52]. TLR-5 appears to play an important role in determining the composition of the gut microbiota during neonatal life. Recent studies show the close relationship between the neonatal TLR-5 expression profile and long-term microbiota selection [53].

NLRs also contribute to modulating the composition of the intestinal microbiota and local homeostasis. The NOD-1 receptor acts as an innate sensor for the formation of adaptive lymphoid tissue and the maintenance of intestinal immune tolerance to commensal microorganisms. The NOD-2 receptor prevents inflammation of the small intestine by limiting the growth of the commensal *Bacteroides vulgatus* [54]. Stimulation of the NOD-2 receptor by commensal bacteria promotes the survival of intestinal stem cells and the regeneration of epithelial cells [55]. The NLRP6 receptor shows tissue and cell-specific expression in the intestinal mucosa. Together with microbial metabolites, it regulates the secretion of IL-18 and antimicrobial peptides by epithelial cells, the mucosal secretion of goblet cells, and is crucial in response to bacteria and viruses [56].

An example of a molecule of microbial derivation with an immunomodulating action is the *polysaccharide A* of the commensal *Bacteroides fragilis*, which, following recognition by TLR-1 and TLR-2, stimulates the expression of genes with anti-inflammatory action, the differentiation of CD4+ T lymphocytes naive in regulatory T lymphocytes (T-reg) and contributes to the maintenance of the Th1/Th2 balance [57]. Another example is butyrate, an SCFA produced by anaerobic bacterial fermentation that can stimulate the differentiation of monocytes into macrophages through the inhibition of histone deacetylase-3 (HDAC3), thus amplifying the host’s innate antimicrobial defense [58].

Finally, recent studies show how the complex phenotypic diversity of innate lymphoid cells (ILCs), essential elements of immunological modulation and tissue repair, can be influenced by the intestinal microbiota [59]. Nongastric *Helicobacter* species can negatively regulate and limit the proliferation of RORγt+ group 3 ILCs, known to be essential elements of immunological control [60]. Group-3 ILCs interact with T-reg causing the deletion of reactive clones towards the commensal flora [61]. Furthermore, group-3 ILCs contribute to the direct maintenance of the commensal flora through the IL-22 axis, limiting the growth of specific microbiota elements [61].

### 3.2. Interactions between the Host Microbiome and Adaptive Immunity

Recent evidence shows that intestinal dysbiosis conditions are also associated with alterations in the mucosal adaptive immune response, both humoral and cell-mediated types [26,27].

One of the most important examples of microbiota regulation of the adaptive immune response is the modulation of secretory immunoglobulin (Ig)-A, which plays a crucial role in protecting the mucosal barriers. The relationship between microbiota and secretory IgA production is mutualistic: on the one hand, the secretory IgA pool contributes to the maintenance of a specific type of commensal microbiome, also avoiding its excessive growth; on the other hand, the presence of specific bacterial species contributes to the production of this IgA family, stimulating the expansion in Peyer’s patches of FoxP3 + T-reg and follicular T-helper (Thf) lymphocytes, which, in turn, promote the differentiation of B lymphocytes in IgA producers [28,62].

The interaction between the intestinal microbiota and CD4+ T lymphocytes is also complex [63]. Some metabolites produced by intestinal bacteria, including SCFAs, may promote the differentiation of CD4+ naïve T cells into T-reg [29]. The Th17 subgroup of CD4+ lymphocytes is known for its dual role in both protection against pathogens and inflammatory disorders. The inflammatory propensity of Th17 is largely determined by the type of intestinal microbiota that induces its differentiation. For example, segmented filamentous bacteria promote, through the ILC-3/IL-22 axis, the development of Th17 RORγt+ with protective activity, while Citrobacter-induced Th17 lymphocytes are a potent source of inflammatory cytokines [28,64].

Another example of microbiota regulation of T lymphocytes is that of CD8+ (cytotoxic) T lymphocytes, whose effector functions are fundamental in the elimination of intracellular pathogens and tumor cells. Intestinal microbial-derived SCFAs are involved in acquiring memory functions by antigen-activated CD8+ T cells [65].

Thf lymphocytes are specialized to assist B cells and are crucial for germinal center formation, affinity maturation, and the generation of high-affinity antibody responses. The relationship between Thf cells and the microbiota is reciprocal, since, if on the one hand, the differentiation of Thf lymphocytes is compromised in germ-free animals, on the other hand, it can be restored by administering TLR-2 receptor agonists to these animals [66].

Finally, a recent randomized, controlled clinical trial in adults with pre-existing impaired immune systems has shown that intestinal dysbiosis induced using broad-spectrum antibiotics is associated with a reduced antibody response to seasonal influenza vaccination [31].

## 4. How Gut and Lung Microbiota Can Influence the Susceptibility to Tuberculosis

**Figure 1:**
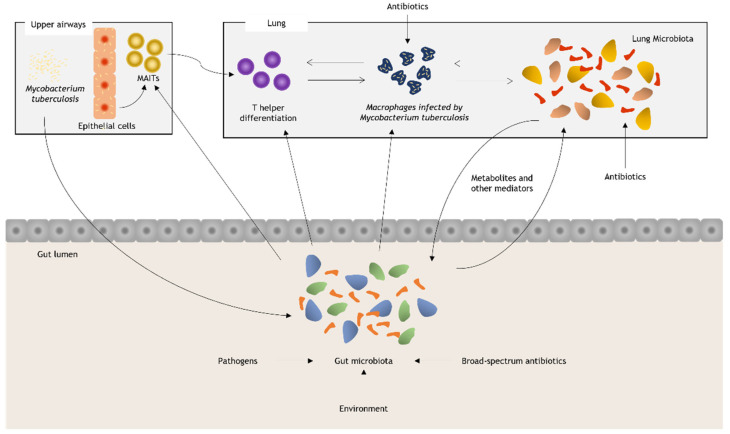
Environmental alterations such as diet, the use of broad-spectrum antibiotics, and colonization by pathogenic bacteria can alter the normal composition of the intestinal microbiota. *M. tuberculosis*, through mostly unknown mechanisms, is also able to modulate the diversity of the intestinal flora. The response of upper pathway epithelial cells and resident invariant T lymphocytes (MAITs) is modulated by the gut microbiota. These can assist the macrophage response to infection. The intestinal microbiota is a strong modulator of the T helper response in the lung and, as such, could affect the ability of macrophages to eliminate *M. tuberculosis* through the increase in the production of IFN-γ, IL-12, and reactive oxygen species. Bacterial-derived metabolites and other mediators are among those responsible for maintaining the dynamic balance between the intestinal and lung microbiota.

Millions of people acquire LTBI each year, and 5–10% of cases develop active TB disease, in some cases without an apparent immunological deficit, suggesting the presence of additional, yet unidentified risk factors [23]. This has led to the hypothesis that an alteration of the gut–lung microbiota axis may be involved in the pathogenesis of *M. tuberculosis* infection and/or in the onset of clinical manifestations of TB. However, a true causal relationship has not yet been well defined (Figure 1).

It has been observed that some important risk factors for TB, such as HIV, malnutrition, diabetes, alcohol, smoking, and pollution, lead to both structural and functional changes in the human microbiota. Cigarette smoking reduces the concentration of some commensal bacteria of the oral cavity (*Porphyromonas*, *Neisseria*, and *Gemella*). At the same time, excessive alcohol consumption causes intestinal dysbiosis (reduction of *Bacteroides* and increase of *Proteobacteria*), which leads to an alteration of permeability intestinal lumen and translocation of metabolites that modulate inflammation [67] (Table 1).

Emerging scientific evidence demonstrates a possible role of the human microbiota in the pathogenesis of TB, response to therapy, clinical outcomes, and post-treatment outcomes. This role is supported by complex multifactorial interactions between the pathogen, the commensal flora, and the host immune response (Figure 1). The major supporting finding of a relationship between human microbiota homeostasis and susceptibility to TB include (1) reduced microbial diversity of gut and lung microbiota is shown in individuals with *M. tuberculosis* respiratory infection compared to healthy controls; (2) susceptibility to *M. tuberculosis* infection and progression to active TB is modified by intestinal coinfection with Helicobacter species; (3) the anaerobes present in the lung coming from the oral cavity by aspiration produce metabolites that can reduce lung immunity and predict the progression of infection to active disease; (4) the increased susceptibility to reinfection of patients who have been previously treated for TB is possibly related to the depletion of antigenic epitopes for T cells in the intestinal commensal microbial flora (nontuberculous mycobacteria); and (5) the prolonged antibiotic treatment used for TB has a long-lasting impact on the composition of gut microbiota [68].

### 4.1. Gut Microbiota and Tuberculosis

The gut microbiota might influence susceptibility to initial *M. tuberculosis* infection and progression from latent infection to active disease (i) by causing interindividual differences in immune cell subsets or their function, both in the gut and in the airways (gut–lung axis); (ii) by influencing the absorption and effectiveness of antibiotic drugs used for the treatment of tuberculosis; and (iii) by producing antimicrobials or immunomodulating molecules that can directly modulate the growth of *M. tuberculosis* [23] (Figure 1).

Despite the accumulating evidence in experimental models, there are still a limited number of human studies assessing the relationship between microbiota and TB. Recent data in animal models and humans show that *M. tuberculosis* infection is associated with alterations in the composition of the intestinal microbiota (decrease in microbial diversity) and metabolic functions. However, these changes are variable between studies and in many cases of minimal magnitude [23,68,69].

In mouse models, a reduction in the relative abundance of the *Clostridiales* (*Lachnospiraceae*, *Ruminococcaceae* families) and *Bacteroidales* orders is observed 6 days after infection with *M. tuberculosis* absence of specific therapy or the detection of mycobacterium DNA in the feces [70].

A recent human study conducted on adults with active pulmonary TB, LTBI, and healthy controls has shown that the active and latent form of TB caused a minor decrease in the alpha diversity of the intestinal microbiota compared to healthy individuals, which mainly resulted from changes in the relative abundance of the genus *Bacteroides* (phylum *Bacteroidetes*) [71].

Depletion of species of phylum *Bacteroidetes* and an increase in species of the phylum *Actinobacteria* and *Proteobacteria* were observed in the gut microbiota of adults with recurrent TB compared to healthy individuals. Furthermore, a reduction of the genus *Lachnospira* (order *Clostridiales*; phylum *Firmicutes*) and the genus *Prevotella* (phylum *Bacteroidetes*) was found in individuals with newly diagnosed active TB and recurrent TB compared to healthy controls. *Lachnospira* and *Prevotella* directly correlated with the number of peripheral CD4+ lymphocytes in patients with newly diagnosed TB and inversely correlated with CD4+ cell counts in individuals with recurrent TB [72].

In another recent study, adults with pulmonary TB showed a distinct stool microbiome, characterized by enriched anaerobes (*Anaerostipes*, *Blautia*, *Erysipelotrichaceae*), before starting the antibiotic treatment. These enriched gut anaerobes correlated with proinflammatory host immune pathways known to be associated with TB disease severity, further supporting the role of gut microbiota in TB pathogenesis [73].

A case-control pediatric study showed that children with pulmonary TB had a decreased microbial diversity in the gut microbiota compared to healthy children. Children with TB had an increased abundance of proinflammatory bacteria of the genus *Prevotella* and the opportunistic pathogen *Enterococcus*, and a decreased abundance of beneficial bacteria of the *Bifidobacteriaceae* family (phylum *Acinbacteria*), *Ruminococcaceae* family, particularly, the SCFAs-producing species *Faecalibacterium ruminococcaceae* and *Faecalibacterium prausnitzii* (order *Clostridiales*; phylum *Firmicutes*), and *Bacteroidaceae* family (phylum Bacteroidetes). Of note, a significant decrease in the gut microbiota richness was observed after a month of antituberculosis therapy. These authors concluded that the homeostasis of gut microbiota may affect the pathogenesis of TB by dysregulation of the hosts’ immune responses through the gut–lung axis [69].

Although human data on the role of the microbiota in mediating initial resistance to *M. tuberculosis* infection are limited, recent data indicate that some commensal bacteria and their antimicrobial products can quantitatively influence initial resistance to various pathogens, such as vancomycin-resistant *Enterococcus*, *Clostridium difficile*, and *Salmonella enterica* [23]. A study on a mouse model has shown that intestinal colonization with the commensal *Helicobacter hepaticus* causes a definite change in the intestinal microbiota, with a prevalence of the *Bacteroides* species and a reduction of *Firmicutes* [74]. This dysbiosis causes an increase in IL-10 and a reduced response to vaccination against *M. tuberculosis* [74]. Another study on germ-free mice has shown that colonization of the digestive tract by *Helicobacter hepaticus* modified the gut microbiota composition and led to impairment of the immune control of the growth of subsequently administered *M. tuberculosis*, which resulted in more significant lung tissue injury [75]. Of note, an experimental study showed that *Helicobacter pylori*, infection of which affects 4.4 million people worldwide, has the opposite relationship with *M. tuberculosis* compared to *Helicobacter hepaticus*, as macaques infected with *Helicobacter pylori* and exposed to aerosol challenge with low-dose *M. tuberculosis* have a lower risk of progressing to active TB compared with uninfected controls [76]. Furthermore, adults with LTBI who do not progress to active TB within 2 years of exposure have a higher likelihood of being infected with *Helicobacter pylori* than those who progress to active TB, suggesting *Helicobacter pylori* can confer immunoprotection against TB [76].

Another plausible mechanism by which commensal metabolites may influence the progression of tuberculous infection is their role in stimulating the abundance and function of bacterial-reactive innate T cell subsets, such as invariant MAIT cells and natural killer T cells (cells iNKT). MAIT cells are indeed absent in germ-free mice, suggesting that their development and function may be influenced by the microbiota [23]. In recent work, it was observed that in adults exposed but not infected with *M. tuberculosis*, resistance to initial infection is accompanied by robust activation of MAIT cells. The levels and function of these cells are correlated with the abundance of specific intestinal microbes [77].

Coinfections during TB can also affect the gut microbiota balance and disease severity. Both children and adults experiencing HIV/TB co-disease tend to have more severe TB course and higher mortality than HIV-negative patients [78]. Reduced diversity and enriched pathobionts have been reported in the gut microbiomes of HIV-infected individuals [79]. HIV infection may also prompt loss of interaction with CD4+ T cells that produce regulatory responses which favor the tolerance of beneficial microbiota [80].

Diet can also affect the composition of the gut microbiota and potentially the immune control of *M. tuberculosis* infection. An experimental study on mouse models has shown that a high-fat diet could trigger a proinflammatory response, with a more rapid progression to active TB [81]. This may be related to intestinal dysbiosis and a reduction in the *Firmicutes/Bacteroidetes* ratio, associated with a decrease in the abundance of the *Porfiromonadaceae* family and, particularly, of the *Barnesiella* genus. It should be noted that a high-fat diet produces an increase in the genera *Alistipes*, *Parasuterella*, *Mucispirillum*, and *Akkermansia*, which have been related to intestinal dysbiotic processes [81].

### 4.2. Lung Microbiota and Tuberculosis

Assessment of the composition of lung microbiota is based on the analysis of sputum or bronchoalveolar lavage (BAL) [82]. Available information on the alteration of the lung microbiota induced by *M. tuberculosis* infection is limited compared to that on gut microbiota, due to the potential contamination of sputum sample with the oropharyngeal flora (e.g., *Prevotella*, *Bulleidia*, and *Atopobium*), the difficulty of sampling lung flora via BAL in affected and healthy individuals, because of the invasive nature of the sampling method, and the lower bacterial biomass contained in the airways [23].

**Figure 2:**
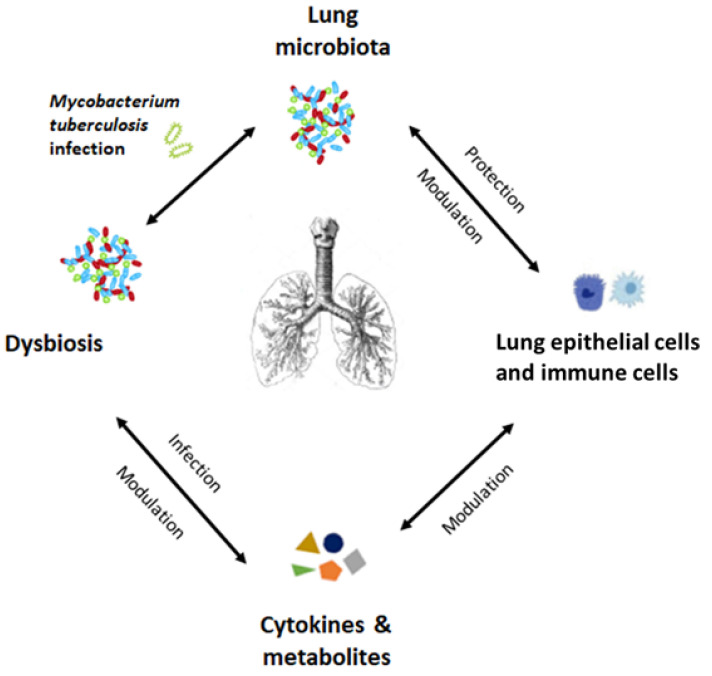
Hypothesized role of the lung microbiota in the pathogenesis of tuberculosis.

Recent evidence shows that individuals with *M. tuberculosis* infection have reduced lung microbiome diversity compared to healthy people. Patients with current *M. tuberculosis* infection have reduced lung microbiome diversity compared to those with prior infection and absence of *M. tuberculosis* in BAL [83] (Figure 2). Although it is difficult to distinguish a common pattern between different studies, individuals with *M. tuberculosis* infection often show enrichment of *Streptococcus* and *Pseudomonas* in the lung microbiota [23,84].

Recent data suggest that the presence of some bacterial strains and changes in the lung microbiota may be associated with the onset of TB and its recurrence and therapeutic failure [85].

Lung microbiota of adults with TB showed a greater abundance of Streptococcus, *Gramulicatella*, *Pseudomonas*, and a lower abundance of *Prevotella*, *Leptotrichia*, *Treponema*, *Catonella*, and *Coprococcus* compared to healthy controls. Subjects with recurrent TB showed a reduced representation of specific genera such as *Bulleidia* and *Atopobium* compared to subjects with new-onset TB [85]. In addition, the *Pseudomonas/M.* ratio in subjects with recurrent TB was lower than in subjects with new *M. tuberculosis* infection, indicating that an imbalance between these bacteria may be a risk factor for recurrence. Pseudomonas was more abundant in the lung microbiota of patients with therapeutic failure than in newly diagnosed and treated TB patients, as was the *Pseudomonas/M.* ratio [85].

Recent evidence has shown a possible role of the lung microbiota in the reactivation process of an LTBI [86]. In a cohort of patients with HIV infections treated with antiretroviral therapy, higher serum concentrations of SCFAs (i.e., propionate and butyrate) were associated with an increased risk of active TB. The increase in SCFAs was associated with an increased abundance of SCFA-producing anaerobic bacteria in the airways, such as *Prevotella*, and with reduced lymphocyte production of IFN-γ and IL-17A in response to *M. tuberculosis*, possibly promoting progression from LTBI to active TB [87].

Furthermore, a pulmonary microbiota enriched with oral commensals, such as *Prevotella*, *Veillonella*, and *Streptococcus*, is associated with an increase in the concentrations of some metabolites (e.g., arachidonic acid) and a proinflammatory phenotype, characterized by the increase in helper T cells that produce IL-17 (Th17) [67]. The microbiota of the lower airways in HIV-positive patients with pneumonia, dominated by *Prevotellaceae*, is an independent predictor of mortality and is also associated with a particular metabolome (enriched with monoglycerides, inosine, and amino acid metabolites) and with a high concentration of proinflammatory cytokines (e.g., IL-17) [67].

### 4.3. Effects of Antituberculosis Therapy on the Human Microbiota

Numerous studies have shown that the administration of antibiotics causes changes in both the intestinal and pulmonary microbiota [88]. The probability of such changes is inversely proportional to the age of exposure and the number and type of antibiotics administered. It can lead to modifications of the host’s immune response to pathogens, favoring the progression and severity of infectious diseases [89]. The recommended first-line therapy regimen for TB involves the oral administration of four antibiotics: isoniazid (INH), rifampicin (RIF), pyrazinamide (PZA), and ethambutol (EMB). The classic therapeutic scheme includes 2 months with INH, RIF, PZA, and EMB (intensive phase), followed by 4 months of INH and RIF (continuation phase). Treatment regimens for drug-resistant TB are protracted (up to 2 years) [90].

As observed in other diseases, the administration of antituberculosis drugs induces profound changes in the human microbiome, which occur both during the therapeutic cycle (acute effects) and after discontinuation of therapy (long-term effects) [23,91]. While such changes on intestinal microbiota are increasingly documented, there are few works related to lung dysbiosis induced by antituberculosis therapy [92,93]. This lack of information is largely related to the diversity of lung conditions that can arise during lung damage (fibrosis, cavitation, bronchostenosis, bronchiectasis, parenchyma alteration) in relation to the age, spread, and severity of the disease, which make it challenging to obtain a homogeneous sample for examination [94].

It has been hypothesized that acute and chronic dysbiosis that occurs during antituberculosis therapy could modulate susceptibility to TB by affecting (i) the microbiome–immune crosstalk, which can affect the course and severity of the disease, (ii) drug metabolism and therapeutic effect, and (iii) risk of reinfection [95,96,97,98].

First-line antituberculosis therapies appear to have a minimal overall effect on intestinal microbiota diversity but modify the relative abundance of specific bacterial taxa [99,100] (Table 2). Subjects treated with antituberculosis therapies show a reduction in the gut microbiota of immunologically critical bacterial species. A common theme is a reduction in the intestinal microbiota of populations of Gram-positive bacteria assigned to the *Ruminococcaceae* and *Lachnospiraceae* family (order *Clostridiales*; phylum *Firmicutes*), which are fundamental in the regulation of homeostasis, in the barrier function of the intestine, the production of SCFAs, and they also regulate the expression of IL-1 and IFN-γ [101]. Other reported modifications in the abundance of bacterial species with immunomodulating activity following anti-TB therapy involve *Bacteroides* spp., which produce polysaccharides that mediate mucosal tolerance through the upregulation of T-reg [102], *Lactobacillus* spp., which modulate the innate and adaptive immune response by binding the PPRs involved in the recognition of bacterial and viral pathogens, *Bifidobacterium* spp., which induce a reduction in the activity of Th17 cells, and *Prevotella*, which is involved in the Th17-mediated inflammatory response [103].

Recent data indicate that, in the first two weeks of antibiotic treatment, resolution of the active inflammatory response of TB (as measured by peripheral blood transcriptomics) may be affected both by *M. tuberculosis* killing, as well as through microbiome-dependent modulation of inflammatory responses. These findings suggest that microbiome perturbation could modify or predict the rapidity of TB resolution [104].

Another aspect of alteration of the host microbiota induced by antituberculosis therapy is the persistence of dysbiosis up to 1–3 years after discontinuation of treatment (chronic effects) [105]. Adults with multidrug-resistant TB showed even longer-term alteration of the gut microbiota, reporting altered taxonomic composition and decrease in richness 3–8 years after recovery and discontinuing the treatment [106].

Markers of persistent dysbiosis are the reduction of the *Clostridiales* of the *Firmicutes* phylum (*clostridiales*, *ruminococcus*, *faecalibacterium*), and an increase in *Actinobacteria* and *Proteobacteria* (*escherichia*, *salmonella*, *yersinia*, *helicobacter*) [67,107]. Of note, recent evidence suggests that the persistence of dysbiosis years after discontinuation of therapy could increase the risk of reinfection [108,109]. A recent study on adults and adolescents suggested that cross-reactivity between certain intestinal microbial species and *M. tuberculosis* epitopes is important for maintaining long-term host resistance to *M. tuberculosis* infection. The effect of antituberculosis therapy on these commensal species could lead to greater susceptibility to reinfection. In this study, the researchers compared the production of IFN-γ in response to different *M. tuberculosis*-related T-cell epitopes by using T cells from patients with or without previous TB. The authors identified a subset of antigenic epitopes (type 2) less effectively recognized by the T cells of subjects previously treated for TB than those without previous TB. These type-2 epitopes were more homologous to bacteria (including nontuberculous mycobacteria) of the gut microbiota than other epitopes. The authors concluded that anti-TB therapy could cause a depletion of the intestinal microbiota necessary to maintain the immune response and the absence of this response could increase the risk of the recurrence of TB [110].

However, to date, the real clinical repercussions of dysbiosis induced by antituberculosis antibiotic therapy are not clear. Moreover, although it is hypothesized that alterations in the intestinal mucosa and its barrier function may be responsible for reduced absorption and drug metabolism, there is limited evidence confirming the importance of dysbiosis on the efficacy of drugs for the therapy of TB [23,111].

## 5. Can Oral Probiotics Be Used in the Treatment of Tuberculosis?

Probiotics are defined by the World Health Organization (WHO) and the Food and Agriculture Organization of the United Nations (FAO) as “live microorganisms which when administered in adequate amounts confer a health benefit on the host” [112]. Probiotics include microorganisms that naturally colonize our mucosal surfaces, including *Lactobacillus* spp., *Bifidobacterium* spp., *Streptococcus salivarius*, and *Escherichia coli* str. Nissle 1917 [113].

The most studied and commonly commercially available probiotic species include *Bifidobacterium* (*adolescentis*, *animalis*, *bifidum*, *breve*, and *longum*) and *Lactobacillus* (*acidophilus*, *casei*, *fermentum*, *gasseri*, *johnsonii*, *reuteri*, *paracasei*, *plantarum*, *rhamnosus*, and *salivarius*). The Consensus of the International Scientific Association for Probiotics and Prebiotics has confirmed that these species can provide general health benefits such as normalization of the altered gut microbiota, regulation of intestinal transit, competitive exclusion of pathogens, and production by SCFA [114].

Furthermore, probiotics can modulate the host’s local and systemic mucosal immune response, interacting with mucosal epithelial cells and with resident cells of innate and adaptive immunity [115]. These complex mechanisms of action likely mimic the natural interactions of the microbiota with the host and may include the induction and/or inhibition of cytokines, chemokines, and antimicrobial peptides, the recruitment or activation of cell populations in the intestinal mucosa, stimulation of the IgA mucosal response, and improvement of barrier and epithelial repair functions [113].

Probiotics can direct the mucosal immune response towards a “tolerogenic” pattern, increasing IL-10 levels [116]. Furthermore, they induce CD4+ Foxp3+ T-reg by inhibiting the production of proinflammatory cytokines and favoring the polarization of T cells towards the Th1 phenotype [117].

The specific cytokine profile depends on the nature and potency of the stimulus, and the strain of probiotic bacteria used [118]. *Lactobacilli* would protect the host from airway infections through interaction with the GALT (e.g., that present in Peyer’s patches) by inducing indirect stimulation of respiratory immune cells [119].

It has also been proposed that the protective effect of probiotics is associated with the activation of NK cells and/or macrophages at the alveolar level. In support of this theory, a study conducted on mouse models has shown that the administration of *Lactobacillus pentosus* increases the activity of NK cells at the splenic level and the production of IFN-γ. The increase of this cytokine occurs through the production of IL-12 by CD11c+ dendritic cells following the interaction between lactobacilli and dendritic cells mediated by TLR2 and TLR4 [120].

Different strains of lactobacilli differ in their ability to induce high levels of IL-12 and consequently of IFN-γ. The effect of probiotics on other inflammatory cells involves T-reg and Th17 lymphocytes in the lung district. Th17s are involved in eliminating pathogens and T-regs at the head of the regulatory processes of immune response in humans and rodents [121].

In an in vitro study, three *Lactobacillus* species (i.e., *casei*, *plantarum*, and *salivarius*), isolated from wild boar feces, showed pH-dependent inhibitory activity against *M. bovis* (responsible for bovine tuberculosis and belonging to the *M. tuberculosis* complex) and influenced its uptake by circulating phagocytes (in which the *M.* survives and replicates) [122]. All lactobacilli demonstrated a significant bactericidal effect at low pH against *M. bovis*, but only *Lactobacillus plantarum* and *Lactobacillus casei* exhibited such antimycobacterial activity at neutral pH. The genomes of the latter revealed the presence of bacteriocins and a collagen adhesion protein with antimycobacterial and immunomodulating action. Furthermore, *Lactobacillus plantarum* significantly reduced macrophage uptake with an antagonistic competition mechanism. The authors concluded by hypothesizing that oral administration of lactobacilli with antimycobacterial activity could reduce the intestinal concentration of *M. bovis* and the risk of its transmission between domestic and wild animals [122].

Other authors have evaluated the use of probiotics for the treatment of multidrug-resistant TB [123]. Gavrilova et al. identified 30 lactobacilli strains capable of inhibiting the growth of *M.b 5* (structurally very similar to *M. tuberculosis* but with less infectious power and therefore suitable for in vitro experiments). In addition, the researchers tested the sensitivity of the identified lactobacilli strains (i.e., *brevis B-3*, *plantarum 22*, *plantarum 2b*, *plantarum 14d*, *cellobiosis 20*, *fermentum 127*, *plantarum 2b* and *14d*, *brevis B-3*, *P. shermanii-15*, *plantarum 22*, *fermentum 127*) to antituberculosis drugs to carry out a possible probiotic–antibiotic combination therapy. All identified probiotic species exhibited sensitivity to rifampicin and resistance to other conventional antituberculosis drugs [123].

Few probiotic strains, including *Lactobacillus* and *Enterococcus* spp., exhibit antibiotic-resistance phenomena. It is hypothesized that the acquisition of antibiotic resistance by some probiotic strains may be disadvantageous since the resistance could spread to other bacteria through the horizontal or vertical transfer of genetic material. This suggests that individual probiotics should be tested for antibiotic-resistance markers before commercialization (although there is currently no evidence of horizontal transfer of antibiotic-resistance genes between probiotics and pathogens) and should ideally be deprived of the plasmid responsible for immunity before using them, to avoid horizontal gene transfer. Another strategy to minimize the risk of inherited resistance could be supplementing antibiotic-resistant probiotics for antibiotic–probiotic combination therapy [124,125].

In a recent study, *Bifidobacterium adolescentis* was shown to exhibit resistance to very high concentrations of rifampicin, to a greater extent than multidrug-resistant *M. tuberculosis* [126]. Mutations in the rpoβ gene cause resistance. The rpoβ gene is a constitutive gene essential for protein synthesis present in almost all prokaryotes and may not be subject to horizontal resistance transfer phenomena. Furthermore, *Bifidobacterium adolescentis* can adapt to Rifampicin concentrations, which could help preserve the human microbiome after treatment with the drug. However, this hypothesis requires other experimental studies [126].

Research conducted on mouse models capable of developing tuberculous lesions similar to human ones has demonstrated the efficacy of a probiotic, *Nyaditum resae^®^* (NR)—containing heat-killed *M. manresensis*—in blocking the development of active TB, through the increase of memory Tregs (CD25+ CD39+) specific for the purified protein derivative [127].

*M. manresensis* belongs to the *M. fortuitum* complex (including nontuberculous bacilli responsible for skin, lymph nodes, and joint infections) and is commonly present in drinking water. A subsequent, double-blind placebo-controlled clinical trial evaluated the safety profile and immunogenicity of the NR probiotic, administered for 14 days to adults, with or without LTBI [128]. In subjects with LTBI, there is an inverse relationship between Th17 and Treg, and the Th17 response can be counterbalanced by the presence of Tregs. An excessive inflammatory response in individuals with LTBI determines the infiltration by Th17 cells, stimulated by the tuberculosis infection itself with intensity dependent on the reactivity of the host [128,129].

The results of this study showed that the administration of the probiotic was able to induce, in both LTBI positive and negative subjects, an increase in effector cells (CD25+ CD39−) and specific memory Tregs (CD25 + CD39+). In conclusion, the study demonstrated that the probiotic NR has a good safety profile and may constitute a new tool to reduce the risk of progression of LTBI towards active TB in humans [128].

## 6. Conclusions

The currently available evidence suggests that the human microbiota might have a role in the pathogenesis of *M. tuberculosis* and that antituberculosis therapy induces short-term and long-term dysbiosis, which can further affect the host immune control of such infection. However, whether changes in the relative abundance of bacterial taxa affect host responses to *M. tuberculosis* infection is currently uncertain due to limited evidence from human studies, which mainly focus on gut microbiota and variable study designs both in animal and clinical models. Further experimental and human research is needed to address the mechanistically cause-and-effect relationship between *M. tuberculosis* and gut and lung dysbiosis. Future research should aim to combine longitudinal analyses, characterizing microbiome changes during *M. tuberculosis* infection, and transcriptome and metabolome profiling, to address whether changes in the relative abundance of any bacterial species lead to biologically meaningful changes in the concentrations of immunomodulatory mediators and metabolites at local and distant tissue sites. Finally, probiotics for the treatment of TB could be a potential option to address the emerging problem of antibiotic resistance, although further studies are needed.

## Figures and Tables

**Table 1:** Risk factors for tuberculosis known to modulate gut or lung microbiota and hepatic antituberculosis drug metabolism (*modified from reference* [67]).

	Intestinal Microbiota	Lung Microbiota	Antituberculosis Drug-Induced Hepatotoxicity
HIV	↓*Rikenellaceae* ↓*Bacteroidaceae* ↓*Lachnospiraceae*	↑*Prevotell,* ↑*Veillonella* ↑*Streptococcus*	Increases risk
Alcohol	↑*Proteobacteria* ↓*Bacteroidetes*	Unknown	Increases risk
Malnutrition	↑*Entreococcus faecalis* ↑*Streptococcus gallolyticus* ↓*Faecalibacterium prauznitzii* ↓*Bacteroides* spp. ↓*Bifidobacterium* spp.	Unknown	Increases risk
Smoking	↑*Proteobacteria* ↑*Bacteroidetes* ↓*Actinobacteria* ↓*Firmicutes*	Minimal effect on microbiota	No data
Air pollution	↑*Firmicutes*, ↓*Bacteroidetes*	↑*Neisseria*, ↑*Streptococcus* ↓*Tropheryma*	Unknown

↑ and ↓ indicate increase and decrease in abundance, respecively.

**Table 2:** Reported modification of the gut microbiota after first-line antibiotic therapy for tuberculosis.

Source	Changes in Microbiota Composition	Reference
Animal (mice)	↑ *Bacteroidetes* *↑* *Clostridiaceae* *↓* *Lachnospiraceae*	[96]
Animal (mice)	↑ *Proteobacteria* ↑ *Bacteroidetes* ↓ *Firmicutes*	[98]
Human	↑ *Bacteroidetes* ↓ *Ruminococcus* ↓ *Faecalibacterium*	[71]
Human	↑ *Fusobacterium* ↑ *Prevotella* ↓ *Blautia* ↓ *Bifidobacterium* ↓ *Firmicutes*	[97]
Animal (mice)	↑ *Bacteroidetes* *↑* *Clostridiaceae* *↓* *Lachnospiraceae*	[96]

↑ and ↓ indicate increase and decrease in abundance, respecively.

## Abbreviations

BAL	bronchoalveolar lavage fluid
EMB	ethambutol
GALT	gut-associated lymphoid tissue
IFN-γ	interferon-gamma
Ig	immunoglobulin
IL	interleukins
ILCs	innate lymphoid cells
INH	isoniazid
LTBI	latent tuberculosis infection
M.	Mycobacterium
MAIT	mucosal-associated T lymphocytes
NLRs	NOD-like receptors
NK	natural killer
PRRs	pattern recognition receptors
PZA	pyrazinamide
RIF	rifampicin
ROI	reactive oxygen intermediates
RNI	reactive nitrogen intermediates
SCFAs	short-chain fatty acids
T-reg	regulatory T lymphocytes
TB	tuberculosis
TLRs	toll-like receptors
Thf	follicular T-helper
TNF-α	tumor necrosis factor-alpha
WHO	World Health Organization
